# The Evaluation of Climate Change Competitiveness via DEA Models and Shannon Entropy: EU Regions

**DOI:** 10.3390/e26090732

**Published:** 2024-08-28

**Authors:** Agnieszka Karman, Jarosław Banaś

**Affiliations:** 1 Institute of Management, Faculty of Economics, Maria Sklodowska Curie University, 20-031 Lublin, Poland; 2 Department of Information Systems and Logistics, Faculty of Economics, Maria Sklodowska Curie University, 20-033 Lublin, Poland; jaroslaw.banas@mail.umcs.pl

**Keywords:** DEA, entropy, optimization, regional competitiveness, climate change

## Abstract

The purpose of this paper is to assess the efficiency of climate change competitiveness via a case study on EU regions by using the data envelopment analysis (DEA) model and Shannon entropy. First, on the same premise as similar composite indicators, we develop a DEA model to assess the relative performance of the regions in climate change competitiveness. Then, we extend our calculations with a DEA-like model and Shannon entropy to derive global estimates of a new competitiveness index by using common weights. Results show that the proposed DEA-Entropy model enables the construction of a regional climate change competitiveness index among all regions via a set of common weights. The proposed model’s common weight structure demonstrates more discriminative power compared to the weights obtained through pure DEA or DEA-like methods. In order to validate the proposed DEA-Entropy model, it was applied to 120 EU regions. The results are meaningful for the regions to improve their competitiveness.

## 1. Introduction

Competitiveness, often associated with productivity, refers to the capacity of a firm, industry, region, or country to achieve a high economic performance by supplying goods and services in a competitive market [1]. Due to its broad conceptual nature, competitiveness has been analyzed both at the microeconomic level, such as firms [2], and the macroeconomic level, such as nations [3]. For regions, competitiveness involves having conditions that allow local companies to excel in their markets, thereby ensuring that the value they create benefits the region [4]. Climate change competitiveness, our focus here, is the region’s ability to achieve and maintain a competitive advantage under the constraints of climate breakdown [5]. Understanding the economic impacts of climate change from a regional economic perspective is crucial. Firstly, because in socio-economic systems, the impacts of climate change are transboundary and not limited to where they occur. Secondly, the model of an economy based on fossil fuels or based on vulnerable sectors, e.g., agriculture, will not be able to continue under climate change conditions due to environmental and social costs. The economic transition will affect the state of the environment, the well-being of local residents, the development of environmentally friendly sectors, and, as a result, the attractiveness of the region. Thus, climate change will significantly influence future social, economic, and environmental outcomes and imply new patterns of regional growth.

Monitoring and the evaluation of the change in regional competitiveness under climate change is carried out on the basis of the Regional Climate Change Competitiveness Index (RCCCI). It is an example of a composite index that has gained increasing popularity due to the fact that it compiles into a single measure a complex phenomenon that a single variable cannot fully capture. In this way, the RCCCI provides a tool for assessing and comparing regional competitiveness, measured as an aggregation of a set of sub-parameters (economic, social, environmental, etc.). Its construction, however, is not straightforward and raises two concerns, analogous to other composite indexes [6], specifically: (1) the index is absolute, making it sensitive to the units of measurement of the parameters, and (2) the selection of weights inherently involves value judgments, meaning that the parameters may not contribute equally to the competitiveness of every region. In order to deal with these concerns, operations research and management science provided a collection of methods to construct composite indexes based on optimization models. Two primary techniques, data envelopment analysis (DEA) and multiple-criteria decision analysis (MCDA), have garnered significant attention as tools for weighting and aggregation in their development [7,8]. For MCDA, additive weighting methods (SAWs) are most commonly used, as well as the uncompensated MCDA method. A key problem of applying MCDA to the construction of the RCCCI is determining the appropriate aggregation of data. The solutions proposed in the literature in this respect, i.e., the weighted product (WP) method, are theoretically superior to the SAW method. However, non-compensatory MCDA approaches are based on the assumption that only the ordinal characteristics of composite indicators are concerned while they are cardinal in nature [9]. As a result, MCDA may lead to less information loss compared to other index construction methods. Another problem is the assignment of weights. Weights are critical components of the RCCCI, as they determine the trade-offs among the chosen parameters. These weights influence the results of regional competitiveness, potentially leading to inaccurate conclusions if subjective criteria are used to determine them. Even with a fixed set of weights, reaching a consensus can be challenging due to the unique characteristics of each entity [6]. To address these issues, a data envelopment analysis approach is recommended.

DEA is the foremost technique for assessing the relative efficiency of decision-making units based on multiple inputs and outputs. The efficiency of a unit is calculated as the weighted sum of its outputs divided by the weighted sum of its inputs, measured on a bounded ratio scale. The superiority of this method is manifested by the fact that it operates without requiring an expert opinion or analyst judgment [10]. The effect of DEA is to classify units into effective and ineffective with the assumption of inefficiency. In this sense, DEA can be used as an optimization approach for a comparative efficiency evaluation.

The DEA method was first applied to regional economics by [11], who used it to evaluate the efficiency of cities in China and proposed that DEA results could help assess the behavior of inefficient regions. Ref. [12] utilized DEA to estimate and monitor the industrial performance of a group of Chinese cities, addressing the evaluation and planning of the economic performance of 28 “key cities” based on three inputs (labor, working funds, and investment) and three outputs (gross industrial output value, profit and taxes, and retail sales) using CCR DEA models. Subsequently, [13] suggested employing DEA to measure the desirability of living in Japan’s 47 prefectures using multiple social indicators, substituting inputs and outputs with negative and positive social indicators, respectively. Ref. [14] continued this approach to study regional performance based on economic–energy–environment variables. Please refer to [15] for the application of DEA for the estimation of how well regions in Serbia utilize their resources, [16] for analyzing the efficiency of the Chinese regional environmental innovation system, and [17] for extending the additive super-efficiency DEA method for evaluating environmental performance. Based on this brief literature review, we can conclude that, while some topics are connected to using DEA to study regional performance, the use of this method to study competitiveness has been scant. Thus, we respond to the call from [18] that data envelopment analysis (DEA) indicates a suitable method for measuring the regional competitiveness score, assuming that the level of regional competitiveness is relative. To the best of our knowledge, besides the studies by [19,20], there appear to be no other similar applications related to regional competitiveness. Moreover, no work addresses regional competitiveness under climate change.

In this paper, the optimization of the regional climate change competitiveness index is considered in light of the selected non-parametric optimization methods. Because the RCCCI provides a good reference for policymakers to estimate a region’s level of competitiveness under climate change, we analyze its effectiveness across EU regions. However, considering the crucial role of the weights in both the calculation of the RCCCI and for the identification of key parameters, two important research questions arise: Given a set of weights and a sample, what is the influence of each parameter on regional climate change competitiveness? How would the level of competitiveness differ if endogenous weights accounting for region heterogeneity were included in the model?

In this paper, the evaluation of the RCCCI is examined through the lens of data envelopment analyses and Shannon entropy. This approach is justified with regard to the RCCCI because: (a) the competitiveness of regions should be compared to the performance of the best regions, and (b) the weights of the parameters should be directly derived from the data. Combining Shannon entropy with DEA efficiency may lead to more accurate and robust outcomes. DEA’s relevance to regional competitiveness assessment lies in its ability to evaluate the relative efficiency of regions in converting inputs into outputs. On the other hand, Shannon entropy is relevant because it quantitatively measures uncertainty, diversity, and unpredictability, key factors in understanding and evaluating competitiveness. Regions face uncertainty regarding regional government plans, investor behavior, and migration movements in a competitive environment. Higher entropy reflects greater uncertainty, which might require more strategic planning and agility to maintain competitiveness. Given the randomness and the many possible variables affecting a region’s situation (and, therefore, competitiveness), entropy can be used for assessing how stable and predictable a region’s competitiveness is. By quantifying these factors, entropy helps policymakers understand the competitive landscape, make informed decisions, and develop regional development strategies.

This paper contributes to optimization methods in three ways. Firstly, compared to the previous research on DEA techniques for optimization, this study advances the literature by applying DEA to assess the efficiency of EU regions to maximize the overall competitiveness score. We propose an approach that uses two sets of weights, region-adjusted and common, which is expected to provide a more reasonable and tailored index. Secondly, we incorporate Shannon entropy into the common weights DEA method to enhance its discriminative power. The entropy weight method assigns objective weights to indicators individually, based on the degree of numerical dispersion among them. Thus, drawing on entropy theory, the proposed model serves as an extension of the basic RCCCI model by utilizing a set of common weights that eliminates subjective bias, thereby making the results more objective and equitable. Thirdly, the use of entropy goes beyond calculation weights; it is about quantifying unpredictability, which allows for a more nuanced assessment of regional competitiveness, in which uncertainty plays a key role. This approach introduces a novel angle by focusing on inherent randomness rather than just deterministic factors. It shifts the focus from just calculating averages to understanding the distribution of possible outcomes. In this way, by applying Shannon entropy to areas traditionally analyzed through deterministic models, it provides a more comprehensive framework for evaluating regional efficiency. Moreover, this paper contributes to regional studies by evaluating regional competitiveness. In this way, a comprehensive framework is proposed to consider various parameters determining competitiveness in climate change conditions.

The paper is organized as follows: In the following section, we describe the method used to calculate the RCCCI (Section 2.1). Next, the mathematical formulations of standard, pure DEA, and DEA-like models are provided, along with various indicators to be used as inputs and outputs (Section 2.2). Furthermore, we provide a detailed formulation of our proposed methodology using Shannon entropy (Section 2.3). Relying on the recent literature, we use Shannon entropy to consolidate various sets of optimal weights into a common set. With this line of thinking, the next section discusses our findings after implementing the methods outlined previously (Section 2.2 and Section 2.3). The paper ends with some concluding remarks.

## 2. Theoretical Background

### 2.1. Regional Climate Change Competitiveness Index

**Figure 1:**
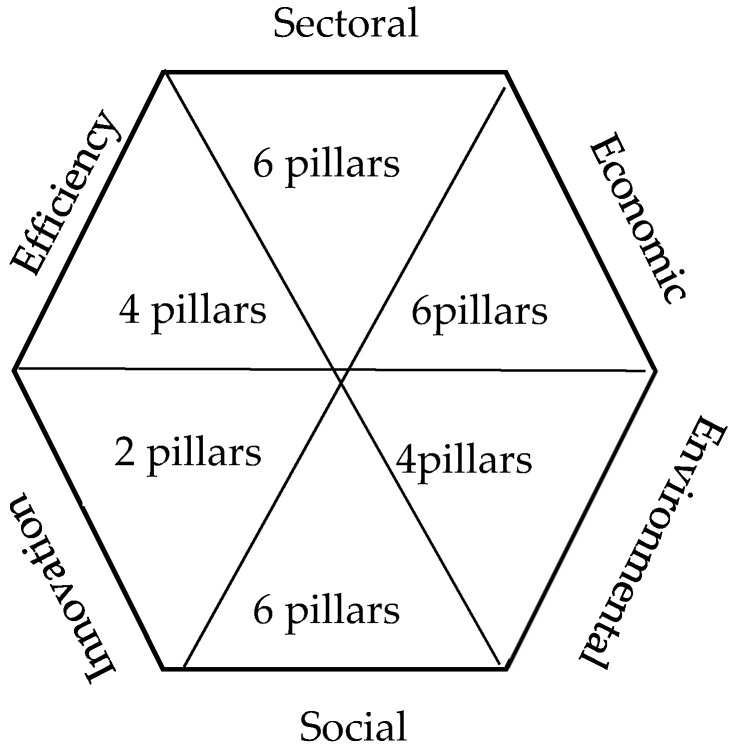
RCCCI framework structure (adapted from [21]).

The RCCCI is an example of a composite indicator. Following [21], it includes six main dimensions of drivers of regional climate change competitiveness: environmental, economic, efficiency, innovation, social, and sectoral (Figure 1).

The environmental dimension covers the factors that identify the state of the environment and the effectiveness of achieving climate goals. Given that natural capital is crucial for development [22], ensuring high environmental quality or implementing measures to achieve it are vital components of the development process, fostering the increase in the competitiveness of the economy, also at the meso-economic level [23]. In turn, the realization of climate targets affects the region in two ways. Firstly, variations in abatement efforts might cause shifts in trade patterns, with companies relocating the production of carbon-intensive goods from countries with stringent regulations to those with looser controls. Consequently, this could result in a loss of employment and reduced economic output in the carbon-intensive sectors of the more rigorously regulated countries [24]. Secondly, according to Porter’s Hypothesis, the efficiency of achieving climate goals favors decoupling by encouraging the adoption of green technologies. In this context, significant economic growth could be accompanied by a decrease in emissions [25].

The economic dimension refers to the key basic drivers of all types of economies. It identifies the main issues that are necessary to develop regional competitiveness, including institutions, infrastructure, and macroeconomic stability. According to the Regional Competitiveness Index [26], we assume that the institutions capture the quality and efficiency of institutions, the level of perceived corruption, and the overall regulatory environment within countries. It indicates whether the institutional climate fosters entrepreneurship and facilitates the process of starting a new business. Infrastructure plays a crucial role in all household activities and economic production. Effective infrastructure not only drives economic growth and enhances quality of life, but also contributes to national security [27]. Similarly, [28] emphasizes that infrastructure is a key factor in regional development, influencing socio-economic activities, regional capacities, and production factors both directly and indirectly. Another important economic factor is economic stability, which encompasses sustainable economic growth, price stability, and employment opportunities [29].

The efficiency dimension groups the efficiency of selected regional subsystems: labor market, market, and resources. It also includes measures of emissions at the regional level. Following [30], the labor market significantly affects both the speed and the region’s overall capacity to adapt to economic changes. Regions with a well-functioning labor market that can retain a skilled workforce contribute to economic growth by creating jobs and adapt more quickly to economic shifts. Moreover, regions with a strong labor market are attractive to businesses looking to expand. Market size determines the extent of the market accessible to businesses. Larger markets enable companies to expand, leverage economies of scale, and potentially drive entrepreneurship and innovation [26]. Tracking regions’ emissions performances and quantifying the driving forces of emissions are of great interest to policymakers since these issues are important to evaluating the effectiveness of policy measures [31]. Reducing emissions suggests that a region incurs fewer environmental costs for the same economic gains [32], which positively drives its development.

Innovation is viewed as the key driver of progress and competitiveness [33], impacting various aspects of human activity, like business, society, science, finance, and technology, and integrating them into a cohesive system [34]. Given that innovation and technology significantly shape living, working, communicating, and entertaining in contemporary society, advancements in these areas largely determine the quality of life for citizens and the economic attractiveness of a region.

The importance of social capital for the region’s competitiveness was highlighted by [35,36], among others. Climate change competitiveness took into account the effects of climate change on society and the awareness and attitudes of residents toward climate change. At the same time, the social dimension includes traditional social indicators of competitiveness, such as the level of social development, and the strength of NGOs and healthcare.

The last dimension of the RCCCI concerns the impact of sectors on climate change and vice versa. It focuses on high-emitting sectors (energy, industry, and transport) and climate-sensitive sectors (agriculture, tourism, and construction). The first group of sectors are significant contributors to anthropogenic global warming, and reducing their emissions could play a significant role in climate change mitigation. The second group covers sectors vulnerable to climate change, where there is significant uncertainty about the impacts of these phenomena, high potential risks, and an urgent need to adapt. The role of the above sectors in shaping competitiveness was highlighted by [37,38,39].

The above dimensions of the RCCCI consist of 28 pillars that stimulate (e.g., biodiversity and achieving climate goals) or inhibit (e.g., industrial sector) competitiveness. Each pillar is determined by indicators that reflect the two-way relationship between climate change and regional development. Thus, anthropogenic impacts on climate change (emissions and waste) and climate change impacts on regions (including health and number of days with extreme temperatures) are included. The full list of pillars and indicators is presented in the Supplementary Materials.

The RCCCI value is the arithmetic sum of normalized pillar values. The position of regions in the RCCCI ranking can be attributed to two primary factors: one is structural, concerning the datum itself, and the other pertains to the specific weighting scheme applied in the RCCCI value estimation. In this paper, we focus on the weighting scheme where the rank of the regions depends strongly on the weights assumed in the RCCCI, and could be reversed if another weighting scheme is selected. Models for estimating the values of the weights are presented in Section 2.2 and Section 2.3.

### 2.2. DEA Model

Data envelopment analysis (DEA) is a nonparametric technique used to assess the relative efficiencies of similar decision-making units (DMUs) by comparing their outputs to their inputs and classifying them as either managerially efficient or inefficient. The method was first introduced by [40] and later popularized by [41]. in 1978. The CCR ratio model developed by [41] aims to optimize the ratio of a linear combination of outputs to a linear combination of inputs. To understand the fundamental concept of a DEA model, consider *n* independent DMUs (regions) whose efficiency (in this case, climate change regional competitiveness) needs to be assessed in relation to each other. Each DMU is evaluated based on *m* input parameters and a given set of *t* output parameters, which are common to all *n* regions. The relative efficiency of a region is measured by how effectively it converts its *m* inputs to *t* outputs. This efficiency is calculated as the ratio of an aggregated output measure to an aggregated input measure. These aggregated measures are obtained by taking non-negative linear combinations of *m* inputs and *t* outputs. Consequently, the input-oriented relative performance (strength or efficiency) *f_j_* of region *j*, *j* = 1, 2, …, *n*, is defined as the maximum value of this ratio, determined over all possible aggregating multipliers, ensuring that no region’s relative performance exceeds unity. Thus, the DEA efficiency measure for a decision-making unit, *j*_0_ ϵ $\left\{1,\;2,\;\ldots ,\;n\right\}$, is derived by solving the following optimization problem:

(1) $$f_{j_{0}}:=max\frac{\sum_{r=1}^{t}u_{r}y_{rj_{0}}}{\sum_{i=1}^{m}v_{i}x_{ij_{0}}}$$

(2) $$\begin{array}{cc} & \left\{u_{r},\;v_{i}\right\}\\ s.t. & \\ & \frac{\sum_{r=1}^{t}u_{r}y_{rj}}{\sum_{i=1}^{m}v_{i}x_{ij}}\;\leq 1\\ & u_{r},\;v_{i}\;\geq 0 \end{array}$$

where:

j=1, 2, …, n—the indices of the decision-making units;
r=1, 2, …, t—the indices of outputs;
i=1, 2, …, m—the indices of inputs;
$y_{rj}$—value of output *r* for unit *j*;
$x_{ij}$—value of input *i* for unit *j*;
$u_{r}$—weight assigned to output *r*;
$v_{i}$—weight assigned to input *i*.

The optimal value of the objective function (1) represents the efficiency measure assigned to unit *j*_0_. To determine the efficiency measures for other decision-making units, similar problems must be solved for each unit individually. Model (1) provides the maximum achievable efficiency for region *j*, denoted as *f_j_*, assuming that every other region uses the same non-negative aggregating multipliers to compute their input-to-output conversion ratios. An efficiency score below 1 suggests that it might be possible to reduce input levels while maintaining the same output, whereas a score of 1 signifies that the region is DEA-efficient. By applying this model to each region separately, we compute the respective maximum relative efficiency score for each one. Although the DEA model described by (1) and (2) is framed as a fractional linear programing problem, it can be conveniently converted into an equivalent linear programing problem:

(3) $${ᵛf}_{j_{o}}:=max\sum_{r=1}^{t}u_{r}y_{rj_{0}}$$

(4) $$\begin{array}{cc} s.t. & \\ & \sum_{r=1}^{t}u_{r}y_{rj}-\sum_{i=1}^{m}v_{i}x_{ij}\leq 0\\ & \sum_{i=1}^{m}v_{i}x_{ij_{0}}=1\\ & u_{r},\;v_{i}\;\geq 0 \end{array}$$

It is easy to show that $ᵛf_{k}=f_{j}$ _j_ holds under the non-negativity of the observed data. More precisely, if $x_{i}>0$ for some i=1, 2, …, m, then $ᵛf_{j}=f_{j}$ holds. In these studies, all the data of this kind are positive.

The dual nature of problems (3) and (4) allows for the identification of a set of efficient units, known as peer units, which demonstrate efficiency using the weights of the inefficient unit. These peer units, associated with the strictly positive basic multipliers in the optimal solution, serve as benchmarks or targets for unit $j_{0}$ (see, for example, [42]).

The efficiency of a certain DMU ($j_{0}=1,\;2,\;\ldots ,\;n$) is defined as follows [41]:

(5) $$h_{j_{0}}=\frac{\sum_{r=1}^{t}u_{rj_{0}}y_{rj_{0}}}{\sum_{i=1}^{m}v_{ij_{0}}x_{ij_{0}}}$$

where ${v=[v_{1},v_{{2j}_{0}},\ldots ,v_{{mj}_{0}}]}^{T}$ and $u{=[u_{1},u_{{2j}_{0}},\ldots ,u_{{tj}_{0}}]}^{T}$ represent the multiplier weights for inputs and outputs, respectively, while $h_{j_{0}}$ is the efficiency of ${DMU}_{j_{0}}$.

A fundamental limitation of the pure DEA model is the inability to rank regions, given that the results are not based on common weights. Thus, in this paper, we additionally use a modified DEA (DEA-like) model to obtain global and, to some extent, indisputable results. To this end, a common set of weights is estimated in such a way that the resulting performance results (global results) are as close as possible to the ideal results, as proposed by [43]. The DEA-like model aims to minimize the total squared distance between the ideal efficiency scores and the efficiency scores derived from the common weights. To assess the proximity between the efficiency measure *E* (*u,v*) and the set of scores {*h_1_, h_2_*, …, *h_n_*} (where each DMU’s efficiency is calculated using model 5), a generalized family of distance measures is applied, as follows:

(6) $$d_{p}\left(E\left(u,v\right)\right)={\left[\sum_{j=1}^{n}(h_{j}-E_{j}{\left(u,v)\right)}^{p}\right]}^{1/p}$$

where *p* represents the distance parameter.

The common weights (*u*, *v*) and the corresponding efficiency score, $E_{j}(u,\;v)$, obtained from the following mathematical program are referred to as the compromise solution with parameter *p*.

(7) $$\begin{array}{cc} & \min d_{p}={\left[\sum_{j=1}^{n}(h_{j}-E_{j}{\left(u,v)\right)}^{p}\right]}^{1/p}\\ \mathrm{s.t.} & \\ & E_{j}(u,\;v)=\sum_{r=1}^{t}u_{r}y_{rj}-\sum_{i=1}^{m}v_{i}x_{ij}\leq 1,\\ & u_{r},\;v_{i}\;\geq 0,\\ & u_{r}\geq 0,\;r=1,\;2,\;\ldots ,t,\\ & v_{t}\geq 0,i=1,2,\ldots ,m \end{array}$$

As noted by [43] for *p* = 2, the objective of model weights $\left(u^{2},\;v^{2}\right)$ is between $E^{2}$ and *h* in the conventional Euclidean space. The common weights in this model are the following:

(8) $$\begin{array}{cc} & \min{\sum_{j=1}^{n}(h_{j}-(\sum_{r=1}^{t}u_{r}y_{rj\;}/\;\sum_{i=1}^{m}v_{t}x_{tj\;}))}^{2}\\ \mathrm{s.t.} & \\ & \sum_{r=1}^{t}u_{r}y_{rj}-\sum_{i=1}^{m}v_{i}x_{ij}\leq 1,\;\;\;j=1,\;2,\;\ldots ,\;n\\ & u_{r}\geq 0,\;r=1,\;2,\;\ldots ,\;t,\\ & v_{i}\geq 0,\;i=1,2,\ldots ,m \end{array}$$

where the optimal solutions ${v=[v_{1},\;v_{2},\;\ldots ,v_{m}]}^{T}$ and ${u=[u_{1},\;u_{2},\;\ldots ,u_{t}]}^{T}$ are the common weights for all DMUs.

### 2.3. Proposed Model with Shannon Entropy

Shannon entropy was introduced by [44]. For a discrete random variable, x, the entropy is defined as:

(9) $$H\left(x\right)=-k\sum_{i=1}^{n}p_{i}{\left(x\right)ln\;p}_{i}(x)$$

where $H\left(x\right)$ denotes the information from each signal source, $p_{i}(x)$ represents the probability of the *i*-th signal source, ${ln\;p}_{i}\left(x\right)$ signifies the information provided by the *i*-th signal source, and *k* is a constant positive integer.

Several researchers have noted that relying solely on the DEA method may lead to unreasonable rankings of DMUs due to its exclusive dependence on input and output indicators, which can limit its discriminative power. However, incorporating the Shannon entropy can enhance both theoretical and practical applications. By integrating the Shannon entropy into the DEA model, we can improve the ranking accuracy, distinguishing the best-performing DMUs from the least efficient ones.

Ref. [45] were the first to incorporate the Shannon entropy into DEA, proposing an entropy DEA model. The core concept of this model is to combine efficiency scores from various DEA models using Shannon entropy. The use of Shannon entropy allows the weights in DEA models to be adjusted, in particular, to account for the distribution and variability of the data. As a result, variables with more information (higher entropy) receive higher weights in the DEA model, whereas those with less variation (lower entropy) are assigned lower weights. Variables with high entropy are considered more informative, as they contribute more to differentiating the DMUs. These variables are assigned higher weights, which reflects their importance in the efficiency analysis.

The process for applying the entropy DEA method, as outlined by [45], involves three steps:

1. Evaluating efficiency using different DEA models;
2. Assessing the importance of each DEA model through Shannon entropy;
3. Aggregating the efficiency scores from the various DEA models.

Therefore, in this paper, to enhance the discriminative capability of the DEA method, we propose a methodology that employs the Shannon entropy to combine various sets of optimal weights into a common set of weights. Then, DMUs can be evaluated using this common set of weights. After [46], we use the following procedure:

1. Calculation of non-zero optimal weights. In this step, we use the DEA model to calculate the non-zero optimal weights, according to Formulas (3) and (4).

Remark: Weight restriction is an effective strategy to prevent zero weights. It has been shown that optimizing these weight restrictions can enhance the discriminative power of the DEA model [47]. Following [46], we estimate initial weights, optimal for each DMU, separately.

Using model (4), we can derive a set of non-zero optimal weights for each DMU. The optimal weights for inputs and outputs are represented by V and U, respectively, as follows:

$$V=\left[\begin{array}{cccc} v_{11} & v_{21} & \ldots & v_{m1}\\ v_{12} & v_{22} & \ldots & v_{m2}\\ \vdots & \vdots & & \vdots \\ v_{1n} & v_{2n} & \ldots & v_{mn} \end{array}\right]\begin{array}{c} \;⃪{DMU}_{1}\\ \;⃪{DMU}_{2}\\ \;\vdots \\ \;⃪{DMU}_{n} \end{array}\;U=\left[\begin{array}{c} u_{11}\\ u_{12}\\ \vdots \\ u_{1n} \end{array}\right]\begin{array}{c} \;⃪{DMU}_{1}\\ \;⃪{DMU}_{2}\\ \;\vdots \\ \;⃪{DMU}_{n} \end{array}$$

2. Compute the values of ${Ȇ}_{i_{1}}$ and ${Ȇ}_{j_{1}}$ (weight normalization). The normalization of the non-zero optimal weights is prepared for the calculation of Shannon entropy.
  (10) $${Ȇ}_{t_{0}j_{0}}^{input}=\frac{v_{t_{0}j_{0}}}{{\sum_{t=1}^{m}v}_{{tj}_{0}}}\;E_{r_{0}j_{0}}^{output}=\frac{u_{r_{0}j_{0}}}{{\sum_{r=1}^{s}u}_{{rj}_{0}}}$$
  Remark: Directly comparing inputs with outputs is inappropriate because these variables are complementary rather than substitutable in DEA models. Therefore, in this step, we separately normalized the non-zero input weights and output weights.
3. Compute entropy *e*_l_ as:
  (11) $$e_{\;j_{0}}^{input}=-e_{0}\;\;\sum_{t=1}^{m}{Ȇ}_{{tj}_{0}\;\;}ln({Ȇ}_{{tj}_{0}})\;\;\;e_{j_{0}}^{output}=-e_{1}\;\;\sum_{r=1}^{s}E_{{rj}_{0}\;\;}ln(E_{{rj}_{0}})\;$$
  where $e_{0}$ and $e_{1}$ are the entropy constants and defined as $e_{0}={(\ln m)}^{-1}$ and $e_{1}={(\ln s)}^{-1}$, respectively. We assume there is always more than one input or output, meaning *m* > 1 or *s* > 1. Especially, the entropy of a single input or single output is assumed to be equal to 0.
4. Calculate the importance degree of optimal weights. Although inputs and outputs may have different practical implications, when translated into Shannon entropies, they both reflect a measure of disorder. As a result, the Shannon entropies of inputs and outputs can be analyzed collectively. The importance degree of a DMU, $j_{0}$ ($j_{0}$= 1, 2, …, n), is defined as follows:
  (12) $$w_{j0}=\frac{e_{j_{0}}^{input}+e_{j_{0}}^{output}}{\sum_{j=1}^{n}e_{j}^{input}+\sum_{j=1}^{n}e_{j}^{output}}$$
  Remark: The degree of importance aligns with maximizing the Shannon entropy. Specifically, the importance degree derived from the Shannon entropy is based on the variance between input weights and output weights.
5. Compute the common weight. ${{v=[v}_{1},\;v_{2},\;\ldots ,\;v_{m}]}^{T}$ and ${u=[u_{1},\;u_{2},\;\ldots ,\;u_{s}]}^{T}$ represent the aggregation of the optimal weights from each DMU with the importance degree as:
  (13) $$v_{t}=\sum_{j=1}^{n}\left(w_{j}v_{tj}\right)\;u_{r}=\sum_{j=1}^{n}\left(w_{j}u_{rj}\right)$$
  where *t* = 1, 2, …, *m* and *r* = 1, 2, …, *s*.

Following these steps, DMUs can be assessed using the common weights \(*v*\) and \(*u*\) as defined by the cross-efficiency approach as follows [48]:

(14) $$k_{j_{1}j_{0}}=\frac{\sum_{r=1}^{s}u_{{rj}_{1}}y_{{rj}_{0}}}{\sum_{t=1}^{m}v_{{tj}_{1}}x_{{tj}_{0}}}$$

where $k_{j_{1}j_{0}}$ is the cross-efficiency of a DMU ($j_{0}=1,\;2,\;\ldots ,\;n$) using the optimal weights of a DMU ($j_{1}=1,\;2,\;\ldots ,\;n$).

## 3. Results

### 3.1. DEA Approaches to the RCCCI

#### 3.1.1. Model and Research Sample

In this paper, we followed [49], and proposed employing the DEA approach to evaluate the relative performance of different regions in terms of regional climate change competitiveness. In this way, we consider regional climate change competitiveness as the output and parameters (pillars) determining its value as inputs. A total of 29 parameters (28 inputs and 1 output) is used, as presented in Table 1. The calculation was carried out for the year 2020. The raw data are presented in the Supplementary Materials.

The reference point for the selection of regions was the typology of climate change regions developed by the ESPON project. From each of the five climate clusters (Northern Europe, Northern Central Europe, Southern Central Europe, Mediterranean region, and North-West Europe), two countries were randomly selected for which all regions at NUTS 2 level were considered. The selected countries included: Bulgaria, Estonia, Finland, Hungary, Ireland, Italy, the Netherlands, Poland, Portugal, and Slovakia. In the end, the research sample covered 120 European regions.

#### 3.1.2. DEA Approach

Let C be the set of 120 randomly selected EU regions of the study, j*ϵC* stand for any region in *C*, and *j*_0_ stand for the evaluated region. Also let *v*_1_, *v*_2_, *… v*_28_ be the unknown weights of the 28 inputs—pillars of the RCCCI. The linear model estimates the weights, *v*, and weight *u_j_* (output = RCCCI) for the region, *j*_0_, solving for one region at a time. The weighted sum of the component indices is restricted to be less than or equal to one for all regions. The pure DEA model that produces the most favorable weights for each region is as follows:

$$\max\;u_{r}y_{{rj}_{0}}$$

Straightforwardly, this model is equivalent to an output-oriented approach. This is in line with [50], who indicates that an output-oriented model in DEA is employed in situations where the primary focus is on maximizing outputs given a fixed level of inputs. When assessing regional competitiveness, the output-oriented model is useful for identifying how many more outputs (competitiveness) can be achieved by the most efficient regions. 

Calculation methods of these parameters are various, so it is better to normalize quantities of parameters to solve this model with the use of DEA. The purpose of normalization is to adjust the size (magnitude) and the relative weighting of the parameters. As a result of normalization, two parameters (Natural_WQ, Efficiency_EEI) were rejected from the model. The variables were removed from the analyses due to large missing data from these sets. Normalization could not be performed correctly for these two areas. Due to space limitations, normalized data are included in the Supplementary Materials.

To perform the analyses, scripts were prepared in Python 3.12 to automate the necessary calculations. The analysis was divided into four stages:

1. Preparation of standardized data.
2. Preparation of models and optimization using the DEA method.
3. Model optimization using the entropy method.
4. Model preparation and DEA-like optimization.

##### Ad. 1. Preparation of Standardized Data

The normalization carried out at the beginning of the analyses was intended to enable comparability between available the data. To ensure that none of the variables had an excessive impact on the others, it was decided to calculate the minimum and maximum values for each of the analyzed pillars and estimate the value according to the following formula:

$$v_{ij}=\frac{V_{ij}-{minV}_{ij}}{maxV_{ij}-minV_{ij}}$$

where:

i=1, 2, …, m—inputs.
j=1, 2, …, n—decision-making units.
$V_{ij}$—values before normalization.
$v_{ij}$—values after normalization.

As a result of these operations, a table was created containing the codes of the studied regions (GEO_code), region names (GEO_labels), values as input parameters (inputs; 26 columns), and RCCCI values (output; single column).

##### Ad. 2. DEA Models and Optimization

**Figure 2:**
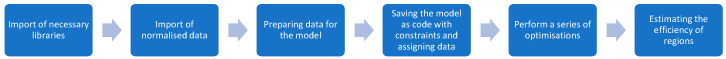
Script diagram for optimization using the DEA method.

For the purpose of optimization, we decided to use Python with additional libraries. The script we used, among others, was the Pyomo library. Thanks to the functionalities available, a model [*model = pyo.ConcreteModel()*] was created, and decision variables from the input [e.g., *model.x0 = pyo.Var(domain = pyo.NonNegativeReals, bounds = (0, None))*] and the output [*model.y = pyo.Var(domain = pyo.NonNegativeReals, bounds = (0, None))*] were defined. To be able to assign specific weight values to the model, normalized data were imported from the file created in the previous step and prepared for the purpose of entering them into the model (the general form of the model). Next, the model [model.OBJ = pyo.Objective(expr = model.y*Current_region_RCCCI, sense = pyo.maximise)] and limiting conditions [*model.limits = pyo.ConstraintList()*; *model.limits.add(…)*] were imported. The optimization was performed using the *glpk* solver. The obtained results were saved to a file for further analysis. A series of optimizations was performed independently (using loops) for each of the analyzed regions. The script outline for the first optimization is shown in the figure below (Figure 2).

##### Ad. 3. Model Optimization Using the Entropy Method

**Figure 3:**
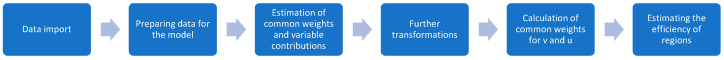
Script diagram for calculating entropy.

Entropy was calculated using the weights determined in the previous step. The sum of the weights was estimated, and then the share of individual weights in their sum was estimated. Values less than 0.000000001 were reduced to 0. For the remaining values, a transformation using the natural logarithm was used. After a series of transformations, the values of common weights (input area; *v*) and common weight (output area, *u*) were obtained. The script outline for calculating entropy is shown in the figure below (Figure 3).

##### Ad. 4. DEA-like Models and Optimization

**Figure 4:**
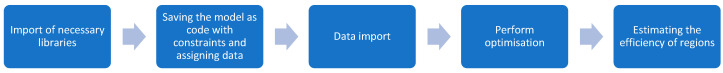
Script diagram for optimization using the DEA-like method.

Another optimization was also carried out using Python, using the Pyomo library. A model [*model = AbstractModel()*] was created, and two areas were defined: Regions and Pillars [*model.Regions = Set()*, *model.Pillars = Set()*]. An important element of constructing the model for optimization was defining the parameters h, x, and y [*model.h = Param(model.Regions)*, *model.x = Param(model.Regions, model.Pillars)*, *model.y = Param(model.Regions)*]. Similarly, as before, the decision variables v and u were defined [*model.v = Var(model.Regions, model.Pillars, within = NonNegativeReals, bounds = (0, 1), initialise = 0.5)*, *model.u = Var(model.Regions, within = NonNegativeReals, bounds = (0, 1), initialize = 0.5)*]. The objective_rule [*def objective_rule(model)*] was defined using an additional function. Similarly, constraints were defined using the function and then added to the model [*model.my_constraint = Constraint(rule = constraint_rule)*]. The dataset (*.dat) [*instance = model.create_instance(data)*] was used for the model. The model was optimized using the *ipopt* solver [*solver_opt = SolverFactory(‘ipopt’)*]. The script diagram for the second optimization is shown in the figure below (Figure 4).

The calculations carried out yielded an optimum value for all regions. The results for a random selection of 15 regions are shown in Table 2. Results for the entire population (120 regions) are summarized in the Supplementary Materials.

The results presented in Table 2 reveal that the optimal solutions from pure DEA produce varying weights. Therefore, caution is needed when interpreting these weights, as different optimal values can lead to divergent conclusions regarding the relative importance of the inputs and outputs. Especially, for some regions, we have optimal solutions with some zero weights for specific pillars. This means that, in these regions, progression in all pillars is not necessary for optimal RCCCI values. For example, the SI04 region (Zahodna Slovenija) scored zero weights for 16 pillars, while the BE10 region (Brussels) presented as many as 18 pillars. Due to the mentioned zero-value weights, this approach directs us toward methods that limit the flexibility in choosing weights—particularly because these zero values result in “effectively ignoring some inputs”, as noted by [51]. Also, from a regional development point of view, obtaining zero weights raises the risk of unbalanced development that ignores certain factors that positively influence regional competitiveness.

The results of the pure DEA method show that the most frequently indicated pillars are infrastructure (71), biodiversity (76), transport (74), building (93), and social development (71). The least frequently indicated pillar was NGO power (10). Thus, the most frequently identified pillars affecting competitiveness under climate change appeared to be those related to the impact of sectors and the state of natural ecosystems.

DEA-like optimization resulted in weight values within a narrow range, ranging from 0.19 to 0.50 (Table 3). For the purposes of the model, it was assumed that the values of the weights should range from 0 to 1 [bounds = (0, 1)], and a value of 0.5 [initialize = 0.5] was used as the starting point.

For seven variables (Basic_Inf, Natural_EiACG, Social_H, Innovation_TR, Innovation_Inn, Efficiency_MS, and Sector_Bui), all obtained weight values are less than 0.499. For another four variables (Basic_IRtCC, Sector_Agr, Sector_Ind, and Sector_Ene), no significantly different values were obtained compared to the starting point. Out of the 120 regions studied, for the Basic_IRtCC and Sector_Agr variables, the values obtained were close to 0.5 in 99.2% of the regions. The same was true for the variable Sector_Ind—94.2% and the variable Sector_Ene—93.3%. Natural_AQ and Social_SD were considered to be the least important factors influencing the competitiveness of the regions, with dominant weights <0.3.

### 3.2. Entropy Approach to the RCCCI

According to the procedure presented in Section 2.3, non-zero optimal weights were calculated in the first step. These weights were taken as the weights obtained from the pure DEA method. These weights were normalized, and the Shannon entropy was calculated from this. The entropy of a single output was defined as 0; see Table 4 for examples. Formula (9) was used to determine the degree of importance of the optimal (common) weights. In order to calculate the common weights, Formula (10) was used (Table 5). According to the Shannon entropy calculations, the most important indicators for regional climate change competitiveness were the impacts from sectors (agriculture, transport, and buildings), infrastructure, and biodiversity. In contrast, the indicators of social development and NGO power had the lowest weight compared to the other indicators.

The resulting weighting values in the analysis using entropy ranged from −0.37 to 0.00 (Table 4). For almost half of the variables analyzed (46%), the values obtained are equal to 0.00 in more than 100 regions (out of 120 regions studied). In the case of seven variables (Market size, Energy, Technological readiness, Industry, Air quality, Attitude, and NGO power), the values obtained were rarely anything other than 0.00. However, there are five variables where the analyzed values are significantly different from zero. These are: Buildings (75.0% of regions), Biodiversity (62.5%), Transport (60.8%), Infrastructure (58.3%), and Effectiveness in achieving climate goals (46.7%). The areas identified are the most important for regional competitiveness.

### 3.3. Efficiency Scores 

To explore the differences between the efficiency of the different regions, as measured by the weights estimated in the previous subsections, we report the efficiency of the RCCCI, which has been derived for the sample through three methods (Table 6).

**Figure 5:**
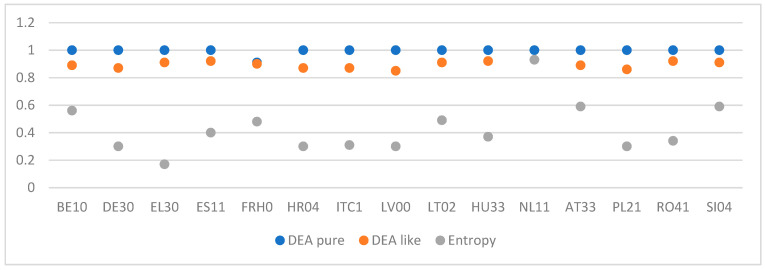
Graphical summary of efficiency results for selected regions.

Firstly, out of the 120 regions, in the DEA pure method, over 100 achieved the highest possible efficiency score (value of 1.00; Table 6). This means that the same level of regional climate change competitiveness could not have been achieved with, among other things, a more negative impact (i.e., sectors) on the environment and a lower efficiency in achieving climate goals, and therefore, these regions have made full use of their conditions (inputs determining regional competitiveness). Secondly, regions in the Netherlands (NL11, NL12, and NL23), along with certain regions in Spain (ES13 and ES23) and Finland (FI19 and FI1D), consistently rank as the top performers across all methods used. Similarly, Dolnoslaskie (PL51), Darmstadt (DE71), and Lazio (ITI4) consistently appear as the lowest-performing regions. In other words, if a region performs exceptionally well or performs poorly across all the RCCCI pillars, its ranking based on the indices will remain consistent, regardless of the method applied. Thus, further optimization methods remain in accordance with the fundamental principles of the original DEA methodology. The next insight consists of observing the value of efficiency. It is evident that the efficiency indices are consistently higher for the pure DEA method compared to the other methods (Figure 5). As Table 6 indicates, a large number of regions achieve a similar score in the DEA models, while the number of efficient regions is significantly reduced in the entropy model. This decrease indicates the increased discriminative power of, i.e., the entropy model compared to the DEA model, since the entropy model seeks the common weights of all regions simultaneously by preventing any individual region from selecting weights that favor it.

**Figure 6:**
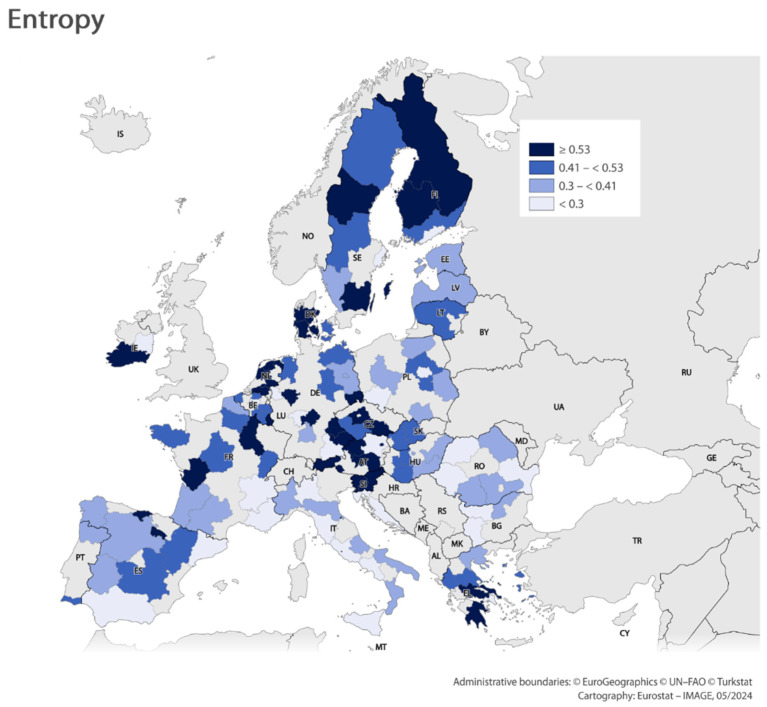
Efficiency scores (entropy). Tool: https://gisco-services.ec.europa.eu/image/screen/home, (accessed on 30 July 2024).

**Figure 7:**
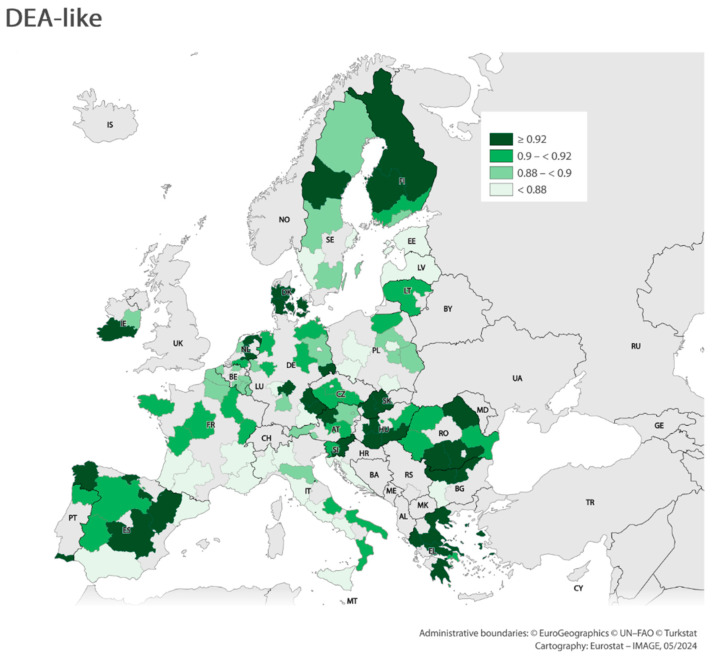
Efficiency scores (DEA-like). Tool: https://gisco-services.ec.europa.eu/image/screen/home, (accessed on 30 July 2024).

Using the results obtained from the three analyses above, maps were made showing regional efficiency scores (classification method: Quantile). Due to the similarity of the values obtained in the pure DEA, these visualizations were made for analyses using entropy (Figure 6) and DEA-like (Figure 7) methods. The values obtained from these analyses are so diverse that they could constitute the basis for preparing such maps. The results indicate that the regions with the highest levels of efficiency are the Nordic regions, the Central European regions, and the Benelux countries. These regions are the main drivers of competitiveness in the EU.

Finally, based on the results of the entropy method, we divided EU regions into three groups—efficient, partly efficient, and inefficient regions. It should be added that, of the 120 regions in the entropy method, only 40 exceeded an efficiency score = 0.5. Following [52], the efficiency scores are classified into three grades: <0.6, 0.6–0.99, and 1.00. The results for the case study are presented in Table 7. The category of the most efficient regions contains Flevoland, Friesland, Groningen, Länsi-Suomi, Niederbayern, Cantabria, and La Rioja.

**Figure 8:**
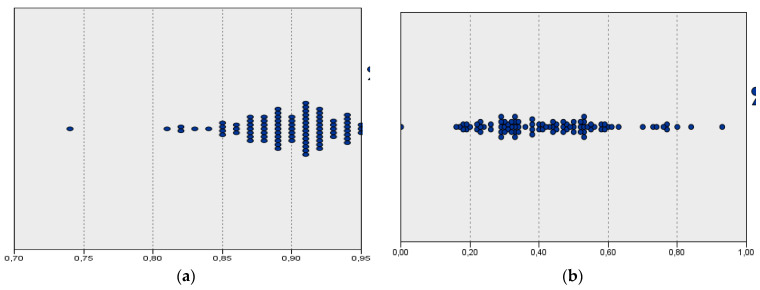
Classification of regions in (**a**) DEA-like and (**b**) entropy methods.

It also should be noted that, with the introduction of arbitrary limits on weights, the efficiency decreases. The efficiency of the regions in the DEA-like method concentrates in the range of 0.85–0.95, while for entropy, the range of 0.2–0.6 dominates (Figure 8). This means that, as the flexibility of the method is reduced, it becomes increasingly difficult to achieve maximum efficiency, despite the increase in discrimination power.

Following [10], the robustness of the proposed method was evaluated using Spearman’s rank correlation test. The Spearman’s rank correlation coefficient for the efficiency values derived from the proposed DEA-like and entropy methods is 0.65. Additionally, the test’s *p*-value is zero, leading to the rejection of H0 at α = 0.00001. This indicates a strong rank correlation between the two sets of RCCCI efficiency values. Consequently, models employing common weights can be regarded as a reliable decision-making tool and a robust alternative to traditional DEA models in developing composite indicators, as previously noted by [53].

## 4. Conclusions

The classic DEA method provides individual regions the maximal flexibility in selecting the weights for inputs and outputs when calculating the efficiency scores. However, this flexibility complicates the creation of a uniform basis for comparison. This paper suggests using the compromise solution approach to establish a common set of weights for the evaluation of the regional competitiveness for a group of regions. First, an ideal value of the composite index—regional climate change competitiveness—was estimated for each region by a DEA index-maximizing model (pure DEA method). The index, based on 28 pillars and 91 indicators, was established to measure regional competitiveness in climate change conditions. In the second stage, a DEA-like model was employed to derive global estimates of regional competitiveness using the common weights. The enhanced superiority of the measurement lies in the optimization process, which renders the weights assigned to component indicators less arbitrary and subject to dispute. Finally, we proposed a comprehensive methodology that integrates DEA with Shannon entropy. The core of our approach is to combine various sets of optimal weights into a unified set using the Shannon entropy. Numerical examples illustrate that each successive method enhances the ability to discriminate RCCCIs more effectively. These examples offer a detailed ranking of EU regions, revealing that some regions perform efficiently, while others do not. A region is considered inefficient if its efficiency score falls below 0.6; our results indicate that 51 EU regions out of 120 fall into this group. These are mainly regions from catching-up countries (Bulgaria and Poland). Taking into account the regions with high efficiency scores (mainly from Germany, Austria, and the Netherlands), it can be expected that they will be the European axis of competitiveness in the coming years. This does not mean that there are no opportunities for other NUTS 2 regions within the European economy at present. However, new growth centers could emerge in Europe. Future outcomes will depend on their ability to use existing resources in the most optimal (efficient) way in order to be more competitive.

This paper compares the different methods both theoretically and through an empirical analysis. These methods can be naturally adapted or expanded by incorporating additional input or output variables, enabling the consideration of other factors pertinent to regional competitiveness comparisons. The evaluation of regional competitiveness is very significant, but its inefficiency is work for the future. In particular, inefficient regions may be a topic for future research. Via the construction of peer units, a strategy for optimal settings of inputs and outputs may be suggested to enhance their competitive positions within the EU. From a methodological point of view, future research could incorporate stochastic noise in competitiveness data and model regional competitiveness by evaluating the efficiencies of regions with a certain probability level.

## Figures and Tables

**Table 1:** Parameters in the optimization model.

Inputs	
Institutions	Basic_Ins
Macroeconomic stability	Basic_MS
Infrastructure	Basic_Inf
Education	Basic_Edu
Institutions related to climate change	Basic_IRtCC
Concentration of economic entities	Basic_CoEE
Water quality	Natural_WQ
Air quality	Natural_AQ
Biodiversity	Natural_Bio
Effectiveness in achieving climate goals	Natural_EiACG
Social development	Social_SD
Awareness	Social_Aw
Attitude	Social_At
Perceived quality of life	Social_PQoF
Health	Social_H
NGO power	Social_NGOP
Technological readiness	Innovation_TR
Innovativeness	Innovation_Inn
Labour market efficiency	Efficiency_LME
Market size	Efficiency_MS
Economic emission intensity	Efficiency_EEI
Resource efficiency	Efficiency_RE
Agriculture	Sector_Agr
Tourism	Sector_Tou
Energy	Sector_Ene
Transport	Sector_Tra
Industry	Sector_Ind
Buildings	Sector_Bui
**Output**	
Regional climate change competitiveness	RCCCI

**Table 2:** Optimal weights for selected regions (pure DEA).

Geo_Code	Geo_Label	Basic_Ins	Basic_MS	Basic_Inf	Basic_Edu	Basic_IRtCC	Basic_CoEE	Natural_AQ	Natural_Bio	Natural_EiACG	Social_SD	Social_Aw	Social_At	Social_PQoL	Social_H	Social_NGOP	Innovation_TR	Innovation_Inn	Efficiency_LME	Efficiency_MS	Efficiency_RE	Sector_Agr	Sector_Tou	Sector_Ene	Sector_Tra	Sector_Ind	Sector_Bui	RCCCI_Weight
BE10	Région de Bruxelles—Capitale	0.03	0.00	2.76	0.58	0.00	0.00	0.00	2.16	0.00	0.00	0.66	0.00	0.00	3.68	0.00	0.00	0.00	0.00	0.00	0.31	0.00	0.00	0.00	0.00	0.00	0.16	0.12
DE30	Berlin	0.09	0.10	0.16	0.00	0.00	0.00	0.00	4.48	1.54	0.00	0.00	0.00	0.00	0.00	0.00	0.00	0.00	0.00	0.00	0.00	3983.50	0.00	0.00	0.00	0.00	0.22	0.11
EL30	Attiki	0.57	0.00	4.67	0.00	0.00	0.00	0.00	1.23	0.00	0.00	0.00	0.00	0.00	0.00	0.00	0.00	0.00	2.29	0.00	0.00	0.00	0.00	0.00	0.00	0.00	3.58	0.16
ES11	Galicia	0.02	0.00	0.00	2.13	0.00	0.00	0.00	0.00	1.61	0.00	0.00	0.00	0.00	0.00	0.00	0.00	0.00	0.00	0.00	0.00	114.60	0.00	0.00	0.00	0.00	0.00	0.13
FRH0	Bretagne	0.00	0.00	0.00	0.23	0.00	0.12	0.00	2.61	1.40	0.00	0.00	0.00	0.15	0.00	0.00	0.00	0.00	0.00	0.00	0.00	3504.09	0.00	0.00	1.06	0.00	0.00	0.11
HR04	Kontinentalna Hrvatska	0.18	0.00	0.32	0.00	0.00	0.80	0.11	9.06	0.00	0.00	0.39	0.00	0.00	0.00	0.00	0.00	0.00	0.00	0.00	0.20	0.00	0.00	0.00	8.15	0.00	0.00	0.18
ITC1	Piemonte	0.01	0.00	0.00	0.34	0.00	0.00	0.00	0.00	0.27	0.10	0.48	0.00	0.00	0.00	0.49	0.00	0.00	0.00	0.00	0.00	148,080.26	0.00	0.00	2.10	0.00	0.00	0.12
LV00	Latvija	0.05	0.00	0.58	0.02	0.00	0.08	0.00	0.00	0.00	0.34	0.71	0.00	0.00	11.92	0.00	0.00	0.00	0.00	0.00	0.21	0.00	0.12	0.00	2.69	0.00	0.23	0.17
LT02	Vidurio ir vakaru	0.00	0.00	0.88	0.00	0.00	0.00	0.00	0.13	0.58	0.21	0.00	0.00	0.00	0.00	0.00	0.00	0.00	0.34	0.00	0.00	0.00	0.00	0.00	14.77	0.00	1.46	0.18
HU33	Dél-Alföld	0.00	0.00	0.40	0.00	0.00	0.00	0.00	0.00	0.00	0.00	0.00	0.00	0.00	0.00	0.00	0.00	1.25	1.84	0.00	0.54	0.00	18.99	0.00	117.43	0.00	1.29	0.17
NL11	Groningen	0.00	0.00	0.26	0.00	0.00	0.00	0.00	5.10	0.00	0.00	0.00	0.00	0.00	0.00	0.00	0.00	0.00	6.10	0.00	0.00	0.00	0.00	0.00	22.88	0.00	2.65	0.11
AT33	Tirol	0.00	0.00	2.17	0.00	0.00	0.00	0.00	0.00	0.74	0.00	0.22	0.00	0.00	0.78	0.00	0.00	0.45	0.00	0.00	0.00	0.00	0.00	0.00	3.67	0.00	1.03	0.13
PL21	Malopolskie	0.00	0.00	0.00	0.11	0.00	0.00	0.05	0.00	0.68	0.00	0.97	0.00	0.00	0.00	0.00	0.00	0.00	0.00	0.00	0.34	1163.63	0.00	17.95	0.00	0.00	0.84	0.19
RO41	Sud-Vest Oltenia	0.00	0.43	1.76	0.14	0.00	0.00	0.00	0.13	0.00	0.00	0.18	0.00	0.00	0.00	0.00	0.00	0.00	0.00	0.00	0.41	0.00	0.00	0.00	18.50	0.00	1.21	0.22
SI04	Zahodna Slovenija	0.22	0.00	0.21	0.00	0.00	0.14	0.00	0.57	0.00	0.27	0.50	0.00	0.00	18.80	0.00	0.00	0.00	0.00	0.00	0.23	0.00	0.00	0.00	2.25	0.00	0.18	0.14

**Table 3:** Optimal weights for selected regions (DEA-like).

Geo_Code	Geo_Label	Basic_Ins	Basic_MS	Basic_Inf	Basic_Edu	Basic_IRtCC	Basic_CoEE	Natural_AQ	Natural_Bio	Natural_EiACG	Social_SD	Social_Aw	Social_At	Social_PQoL	Social_H	Social_NGOP	Innovation_TR	Innovation_Inn	Efficiency_LME	Efficiency_MS	Efficiency_RE	Sector_Agr	Sector_Tou	Sector_Ene	Sector_Tra	Sector_Ind	Sector_Bui	RCCCI_Weight
BE10	Région de Bruxelles—Capitale	0.28	0.19	0.47	0.36	0.50	0.22	0.19	0.48	0.33	0.30	0.31	0.27	0.24	0.50	0.27	0.38	0.45	0.49	0.49	0.27	0.50	0.46	0.50	0.49	0.50	0.24	0.29
DE30	Berlin	0.25	0.28	0.42	0.36	0.50	0.33	0.25	0.49	0.30	0.23	0.25	0.24	0.24	0.47	0.25	0.31	0.33	0.37	0.49	0.37	0.50	0.41	0.50	0.40	0.50	0.33	0.28
EL30	Attiki	0.40	0.21	0.45	0.42	0.50	0.48	0.25	0.49	0.20	0.30	0.20	0.31	0.50	0.47	0.35	0.38	0.37	0.50	0.50	0.48	0.50	0.50	0.50	0.32	0.50	0.44	0.40
ES11	Galicia	0.32	0.29	0.36	0.50	0.50	0.36	0.25	0.31	0.26	0.19	0.20	0.21	0.26	0.49	0.38	0.39	0.41	0.47	0.50	0.39	0.50	0.48	0.50	0.48	0.50	0.45	0.32
FRH0	Bretagne	0.25	0.28	0.36	0.37	0.50	0.38	0.25	0.48	0.30	0.23	0.24	0.26	0.25	0.48	0.28	0.40	0.40	0.44	0.49	0.30	0.50	0.47	0.50	0.48	0.50	0.31	0.10
HR04	Kontinentalna Hrvatska	0.39	0.34	0.29	0.30	0.50	0.46	0.50	0.48	0.23	0.23	0.32	0.32	0.34	0.47	0.39	0.39	0.43	0.38	0.50	0.48	0.50	0.48	0.50	0.48	0.50	0.36	0.44
ITC1	Piemonte	0.36	0.23	0.26	0.40	0.50	0.33	0.25	0.32	0.23	0.25	0.23	0.32	0.30	0.47	0.37	0.38	0.41	0.42	0.49	0.31	0.50	0.46	0.50	0.48	0.50	0.34	0.31
LV00	Latvija	0.32	0.39	0.34	0.37	0.50	0.28	0.19	0.47	0.23	0.42	0.50	0.38	0.34	0.49	0.42	0.41	0.34	0.44	0.50	0.34	0.50	0.48	0.50	0.46	0.50	0.34	0.41
LT02	Vidurio ir vakaru	0.30	0.39	0.41	0.38	0.50	0.35	0.19	0.48	0.34	0.34	0.32	0.31	0.32	0.49	0.37	0.41	0.43	0.46	0.50	0.40	0.50	0.49	0.50	0.49	0.50	0.39	0.45
HU33	Dél-Alföld	0.36	0.36	0.36	0.40	0.50	0.41	0.25	0.38	0.30	0.27	0.22	0.26	0.32	0.49	0.44	0.42	0.48	0.50	0.50	0.44	0.50	0.50	0.50	0.50	0.50	0.29	0.41
NL11	Groningen	0.22	0.30	0.43	0.36	0.50	0.40	0.19	0.49	0.25	0.19	0.30	0.26	0.21	0.50	0.19	0.37	0.48	0.49	0.49	0.24	0.50	0.50	0.50	0.50	0.50	0.37	0.28
AT33	Tirol	0.23	0.25	0.45	0.38	0.50	0.41	0.25	0.42	0.32	0.23	0.33	0.25	0.20	0.49	0.26	0.39	0.45	0.46	0.49	0.37	0.50	0.31	0.50	0.49	0.50	0.36	0.32
PL21	Malopolskie	0.32	0.38	0.38	0.35	0.50	0.35	0.50	0.47	0.32	0.25	0.34	0.41	0.25	0.47	0.38	0.39	0.41	0.44	0.50	0.42	0.50	0.47	0.50	0.47	0.50	0.33	0.47
RO41	Sud-Vest Oltenia	0.44	0.42	0.39	0.46	0.50	0.37	0.25	0.40	0.22	0.24	0.40	0.50	0.32	0.48	0.50	0.43	0.47	0.47	0.50	0.50	0.50	0.50	0.50	0.50	0.50	0.36	0.55
SI04	Zahodna Slovenija	0.31	0.30	0.40	0.36	0.50	0.41	0.25	0.45	0.24	0.29	0.28	0.21	0.27	0.49	0.24	0.39	0.46	0.46	0.50	0.34	0.50	0.45	0.50	0.49	0.50	0.40	0.34

**Table 4:** Shannon entropy and importance degree.

Geo_Code	Geo_Label	Basic_Ins	Basic_MS	Basic_Inf	Basic_Edu	Basic_IRtCC	Basic_CoEE	Natural_AQ	Natural_Bio	Natural_EiACG	Social_SD	Social_Aw	Social_At	Social_PQoL	Social_H	Social_NGOP	Innovation_TR	Innovation_Inn	Efficiency_LME	Efficiency_MS	Efficiency_RE	Sector_Agr	Sector_Tou	Sector_Ene	Sector_Tra	Sector_Ind	Sector_Bui	Output_Entr	Imp_Degree
BE10	Région de Bruxelles—Capitale	−0.02	0.00	−0.35	−0.16	0.00	0.00	0.00	−0.33	0.00	0.00	−0.18	0.00	0.00	−0.37	0.00	0.00	0.00	0.00	0.00	−0.11	0.00	0.00	0.00	0.00	0.00	−0.06	0.00	0.02
DE30	Berlin	0.00	0.00	0.00	0.00	0.00	0.00	0.00	−0.01	0.00	0.00	0.00	0.00	0.00	0.00	0.00	0.00	0.00	0.00	0.00	0.00	0.00	0.00	0.00	0.00	0.00	0.00	0.00	0.00
EL30	Attiki	−0.14	0.00	−0.37	0.00	0.00	0.00	0.00	−0.23	0.00	0.00	0.00	0.00	0.00	0.00	0.00	0.00	0.00	−0.31	0.00	0.00	0.00	0.00	0.00	0.00	0.00	−0.36	0.00	0.02
ES11	Galicia	0.00	0.00	0.00	−0.07	0.00	0.00	0.00	0.00	−0.06	0.00	0.00	0.00	0.00	0.00	0.00	0.00	0.00	0.00	0.00	0.00	−0.03	0.00	0.00	0.00	0.00	0.00	0.00	0.00
FRH0	Bretagne	0.00	0.00	0.00	0.00	0.00	0.00	0.00	−0.01	0.00	0.00	0.00	0.00	0.00	0.00	0.00	0.00	0.00	0.00	0.00	0.00	0.00	0.00	0.00	0.00	0.00	0.00	0.00	0.00
HR04	Kontinentalna Hrvatska	−0.04	0.00	−0.07	0.00	0.00	−0.13	−0.03	−0.35	0.00	0.00	−0.08	0.00	0.00	0.00	0.00	0.00	0.00	0.00	0.00	−0.05	0.00	0.00	0.00	−0.36	0.00	0.00	0.00	0.01
ITC1	Piemonte	0.00	0.00	0.00	0.00	0.00	0.00	0.00	0.00	0.00	0.00	0.00	0.00	0.00	0.00	0.00	0.00	0.00	0.00	0.00	0.00	0.00	0.00	0.00	0.00	0.00	0.00	0.00	0.00
LV00	Latvija	−0.02	0.00	−0.12	−0.01	0.00	−0.03	0.00	0.00	0.00	−0.08	−0.13	0.00	0.00	−0.25	0.00	0.00	0.00	0.00	0.00	−0.05	0.00	−0.04	0.00	−0.29	0.00	−0.06	0.00	0.01
LT02	Vidurio ir vakaru	0.00	0.00	−0.15	0.00	0.00	0.00	0.00	−0.04	−0.11	−0.05	0.00	0.00	0.00	0.00	0.00	0.00	0.00	−0.07	0.00	0.00	0.00	0.00	0.00	−0.18	0.00	−0.20	0.00	0.01
HU33	Dél-Alföld	0.00	0.00	−0.02	0.00	0.00	0.00	0.00	0.00	0.00	0.00	0.00	0.00	0.00	0.00	0.00	0.00	−0.04	−0.06	0.00	−0.02	0.00	−0.27	0.00	−0.16	0.00	−0.04	0.00	0.01
NL11	Groningen	0.00	0.00	−0.04	0.00	0.00	0.00	0.00	−0.27	0.00	0.00	0.00	0.00	0.00	0.00	0.00	0.00	0.00	−0.30	0.00	0.00	0.00	0.00	0.00	−0.30	0.00	−0.19	0.00	0.01
AT33	Tirol	0.00	0.00	−0.34	0.00	0.00	0.00	0.00	0.00	−0.20	0.00	−0.09	0.00	0.00	−0.21	0.00	0.00	−0.15	0.00	0.00	0.00	0.00	0.00	0.00	−0.37	0.00	−0.25	0.00	0.02
PL21	Malopolskie	0.00	0.00	0.00	0.00	0.00	0.00	0.00	0.00	0.00	0.00	−0.01	0.00	0.00	0.00	0.00	0.00	0.00	0.00	0.00	0.00	−0.02	0.00	−0.06	0.00	0.00	−0.01	0.00	0.00
RO41	Sud-Vest Oltenia	0.00	−0.08	−0.20	−0.03	0.00	0.00	0.00	−0.03	0.00	0.00	−0.04	0.00	0.00	0.00	0.00	0.00	0.00	0.00	0.00	−0.07	0.00	0.00	0.00	−0.17	0.00	−0.16	0.00	0.01
SI04	Zahodna Slovenija	−0.04	0.00	−0.04	0.00	0.00	−0.03	0.00	−0.09	0.00	−0.05	−0.08	0.00	0.00	−0.18	0.00	0.00	0.00	0.00	0.00	−0.04	0.00	0.00	0.00	−0.23	0.00	−0.04	0.00	0.01

**Table 5:** Common weights.

Parameter	Weight	Parameter	Weight
Basic_Ins	0.06	Social_NGOP	0.00
Basic_MS	0.03	Innovation_TR	0.03
Basic_Inf	1.50	Innovation_Inn	0.20
Basic_Edu	0.17	Efficiency_LME	1.78
Basic_IRtCC	778.25	Efficiency_MS	0.21
Basic_CoEE	0.15	Efficiency_RE	0.15
Natural_AQ	0.01	Sector_Agr	2971.51
Natural_Bio	2.19	Sector_Tou	0.48
Natural_EiACG	0.25	Sector_Ene	0.43
Social_SD	0.03	Sector_Tra	10.07
Social_Aw	0.22	Sector_Ind	1.58
Social_At	0.01	Sector_Bui	1.74
Social_PQoF	0.02	RCCCI_y	0.15
Social_H	3.95		

**Table 6:** Results of optimizations.

Geo_Code	Geo_Label	DEA Pure	DEA-Like	Entropy
BE10	Région de Bruxelles—Capitale	1.00	0.89	0.56
DE30	Berlin	1.00	0.87	0.30
EL30	Attiki	1.00	0.91	0.17
ES11	Galicia	1.00	0.92	0.40
FRH0	Bretagne	0.91	0.9	0.48
HR04	Kontinentalna Hrvatska	1.00	0.87	0.30
ITC1	Piemonte	1.00	0.87	0.31
LV00	Latvija	1.00	0.85	0.30
LT02	Vidurio ir vakaru Lietuvos	1.00	0.91	0.49
HU33	Dél-Alföld	1.00	0.92	0.37
NL11	Groningen	1.00	0.93	0.93
AT33	Tirol	1.00	0.89	0.59
PL21	Malopolskie	1.00	0.86	0.30
RO41	Sud-Vest Oltenia	1.00	0.92	0.34
SI04	Zahodna Slovenija	1.00	0.91	0.59

**Table 7:** Classification of regions according to entropy-based optimization.

Grade	Grade Centres	Number of Regions	Examples
1	0.77	16	DE22, NL32, AT33
2	0.49	53	BE10, BE35, CZ06, DE80, IE05, ES24, FRH0
3	0.27	51	BG31, DE21, ITG1, PL21

## Data Availability

All the new data obtained in this research are contained in the article or in the Supplementary Materials (available at: https://zenodo.org/records/12167529, accessed on 30 July 2024).

## Supplementary Materials

The following supporting information can be downloaded at: https://zenodo.org/records/12167529 (accessed on 30 July 2024).
